# Targeting Wild-Type and Mutationally Activated FGFR4 in Rhabdomyosarcoma with the Inhibitor Ponatinib (AP24534)

**DOI:** 10.1371/journal.pone.0076551

**Published:** 2013-10-04

**Authors:** Samuel Q. Li, Adam T. Cheuk, Jack F. Shern, Young K. Song, Laura Hurd, Hongling Liao, Jun S. Wei, Javed Khan

**Affiliations:** Oncogenomics Section, Pediatric Oncology Branch, Center for Cancer Research, National Cancer Institute, National Institutes of Health, Bethesda, Maryland, United States of America; University of Pittsburgh Cancer Institute, United States of America

## Abstract

Rhabdomyosarcoma (RMS) is the most common childhood soft tissue sarcoma. Despite advances in modern therapy, patients with relapsed or metastatic disease have a very poor clinical prognosis. Fibroblast Growth Factor Receptor 4 (FGFR4) is a cell surface tyrosine kinase receptor that is involved in normal myogenesis and muscle regeneration, but not commonly expressed in differentiated muscle tissues. Amplification and mutational activation of FGFR4 has been reported in RMS and promotes tumor progression. Therefore, FGFR4 is a tractable therapeutic target for patients with RMS. In this study, we used a chimeric Ba/F3 TEL-FGFR4 construct to test five tyrosine kinase inhibitors reported to specifically inhibit FGFRs in the nanomolar range. We found ponatinib (AP24534) to be the most potent FGFR4 inhibitor with an IC_50_ in the nanomolar range. Ponatinib inhibited the growth of RMS cells expressing wild-type or mutated FGFR4 through increased apoptosis. Phosphorylation of wild-type and mutated FGFR4 as well as its downstream target STAT3 was also suppressed by ponatinib. Finally, ponatinib treatment inhibited tumor growth in a RMS mouse model expressing mutated FGFR4. Therefore, our data suggests that ponatinib is a potentially effective therapeutic agent for RMS tumors that are driven by a dysregulated FGFR4 signaling pathway.

## Introduction

Rhabdomyosarcoma (RMS) is the most common soft tissue sarcoma in childhood, accounting for about 3% of all childhood tumors [1]. Treatment of RMS includes the use of intensive chemotherapeutic regimens in combination with surgical and radiation therapy. This strategy has improved the survival rate for patients with localized disease to 70% albeit with significant toxicity [2]. Despite aggressive multimodal therapy, high risk patients continue to have a poor prognosis with overall survival rates of 20–30% [3]. Therefore, there remains a great need for new therapies targeting the molecular pathways which are found to be altered in RMS.

RMS tumors typically arise from skeletal muscle and are categorized as either of the alveolar (ARMS) or embryonal (ERMS) subtype based on their histology. ARMS tumors are driven by a translocation involving chromosome 2 or 1 with chromosome 13, resulting in the production of the fusion oncogene *PAX3-* or *PAX7-FOXO1*, respectively [4]. In contrast, ERMS tumors commonly harbor loss of heterozygosity at 11p15.5 [5] as well as point mutations in *TP53* [6], *NRAS, KRAS, HRAS* [7], *PIK3CA* [8] and *FGFR4* [9] genes.

Fibroblast Growth Factor Receptor 4 (FGFR4), a FGF receptor family member, is a receptor tyrosine kinase that is implicated in the differentiation of myoblasts into skeletal muscle [10] and muscle regeneration after injury [11]. Highlighting a potential role in RMS, early microarray studies of RMS cell lines and tumors showed massive overexpression of *FGFR4* [12] and subsequent work showed that *FGFR4* is a direct transcriptional target of the PAX3-FOXO1 fusion protein [13]. Of note, recent sequencing studies identified activating mutations specific to *FGFR4* in 7.5% of RMS tumors. These mutations occur at amino acid 535 and 550 of the kinase domain and promote tumor growth and metastasis *in vivo* by constitutively activating FGFR4 [9]. These reports emphasize the importance of FGFR4 in RMS and establish this cell surface tyrosine kinase receptor as a candidate target for RMS therapy.

Ponatinib is an orally administered tyrosine kinase inhibitor that was initially developed as an inhibitor for native and mutant forms of BCR-ABL [14]. Recently, this therapy received accelerated FDA approval for the treatment of adult patients with Philadelphia chromosome positive acute lymphoblastic leukemia (Ph+ ALL) and chronic phase, accelerated phase, or blast phase chronic myeloid leukemia (CML) who are resistant or intolerant to prior tyrosine kinase inhibitor therapy. The inhibition profile of ponatinib includes several other tyrosine kinases, including FLT3, SRC, KIT, PDGFR, and FGFR [14], [15]. Of note, ponatinib has been shown to inhibit all four members of the FGFR family with an IC_50_ of less than 40 nM [16]. Inhibition of FGFR family members by ponatinib has been demonstrated in preclinical models of endometrial cancers with FGFR2 mutations, bladder cancers with FGFR3 mutations, as well as breast, lung, and colon cancer cell lines harboring amplification of the *FGFR1* or *FGFR2* gene [16]. In this study, a panel of RMS cell lines as well as a Ba/F3 cell line engineered to overexpress FGFR4 were tested for sensitivity to five FGFR tyrosine kinase inhibitors, including AP24534 (ponatinib), AZD2171 (Cediranib), BIBF1120 (Nintedanib), TKI258 (Dovitinib), and PHA739358 (Danusertib). Of these, ponatinib was found to be the most potent FGFR4 inhibitor, inhibiting both wild-type and mutated FGFR4 phosphorylation and cell growth. Ponatinib also inhibited growth of tumors expressing mutated FGFR4 *in vivo*. Therefore, our results indicate that ponatinib is an effective FDA-approved drug which has the potential to treat RMS with overexpressed or mutated FGFR4.

## Materials and Methods

### Ethics Statement

This study is compliant with the animal care and experimental procedures which were approved by the National Institutes of Health Animal Care and Use Committee (Proposal Number: PB-038).

### Cell Culture

All RMS772 transfected cell lines were established and maintained as previously described [9], [17]. RMS cell lines were all grown in either RPMI-1640 (RH28, JR, RH18, RD, CTR, BIRCH, TTC-516, and TTC-442) or DMEM medium (RD, RH30, RH4, RH5, RH41, and RH36) (Quality Biological) supplemented with 10% FBS (Hyclone), 2 mM L-glutamine (Quality Biological), and 1% penicillin/streptomycin (Quality Biological). All RMS cell lines were previously established [18], [19] and kind gifts from Dr. Timothy Triche (Children's Hospital of Los Angeles). Ba/F3 cells (RCB0805; Riken BRC) were cultured in RPMI-1640 medium supplemented with 10% FBS, 2 mM L-glutamine, 1% penicillin/streptomycin, and 10% of WEHI-3BD conditioned medium, which contains IL-3. CRL-7250 and U2-OS cells (American Type Culture Collection) were grown in DMEM supplemented with 10% FBS, 2 mM L-glutamine, and 1% penicillin/streptomycin. A4573 cells were kind gifts from Dr. Todd Waldman (Georgetown University) and were grown in RPMI-1640 medium supplemented with 10% FBS, 2 mM L-glutamine, and 1% penicillin/streptomycin, as previously described [20].

### Drug-dose Response Assay and IC_50_ Calculations

We measured relative cell number with the CellTiter-Glo assay (Promega) or relative percent confluency with the IncuCyte (Essen Bioscience). In brief, cells were seeded overnight in 180 µL of culture medium per well in 96-well plates so that they would reach 80% confluency by the end of the assay. After overnight incubation, 20 µL of culture medium containing the various inhibitors was added. Relative cell number was measured at 24 hour intervals after the addition of drug. Prism (GraphPad Software) was used for curve fitting and calculating IC50s.

### Quantitative RT-PCR

RNA was extracted using the AllPrep DNA/RNA Mini Kit (Qiagen). RNA integrity number (RIN) was calculated using the RNA 6000 Nano Kit (Agilent) and all were greater than 9.0. Quantitative RT-PCR using Taqman assays (FGFR4: Hs00242558_m1 and GAPDH: Hs99999905_m1) on a Fluidigm system was previously described [21]. Briefly, cDNA was generated from 200 ng of RNA using reverse transcription. Then, PCR was carried out on a 48.48 Dynamic Array using the BioMark HD real-time PCR system (Fluidigm). Twelve replications were performed for each gene and sample, and average threshold cycle numbers were calculated. FGFR4 gene expression levels were represented by normalizing against GAPDH.

### Cell Cycle Assay

The FITC BrdU Flow Kit (BD Biosciences) was used for cell cycle analysis. In a T25 flask, 1 million cells were seeded and incubated overnight. Cells were treated with ponatinib at a final concentration of 0, 0.625, 1.25, or 2.5 µM for 24 hours. Then the cells were pulsed with 1 mM BrdU for 30 minutes and stained with an anti-BrdU antibody, followed by 7-AAD staining per the manufacturer's guidelines. FACS data was analyzed using CellQuest software (BD Biosciences).

### Caspase-3/7 Assay

In an opaque, flat-bottom 96-well plate, 5,000 cells were seeded in each well with 80 µL of culture medium. After overnight incubation, 20 µL of culture medium containing ponatinib was added to reach final concentrations of 0, 1.25, 2.5, and 5 µM. After 6 hours, ApoLive-Glo (Promega) was used to measure caspase-3/7 activity per the manufacturer's protocol.

### Immunoblotting

RH4, RH5, CTR, RH41, RMS772/FGFR4 (N535K), and RMS772/FGFR4 (V550E) cell lines were treated with ponatinib at 0, 200, and 800 nM for 8 hours. Cells were lysed in RIPA buffer (150 mM NaCl, 25 mM Tris•HCl pH 7.6, 0.1% SDS, 1% sodium deoxycholate, 1% NP-40) with 1% Halt Protease and Phosphatase Inhibitor Cocktail (Thermo). For FGFR4 autophosphorylation immunoblots, 200–500 µg of protein lysate, as determined by BCA protein assay (Pierce), was first immunoprecipitated with a FGFR4 antibody (sc-124; Santa Cruz Biotechnology) and then incubated overnight with protein A/G agarose beads. Proteins were resolved by SDS-PAGE in a 4–12% Bis-Tris Gel (Invitrogen) and transferred to a nitrocellulose membrane by the iBlot (Invitrogen). Membranes were blocked with 5% nonfat dry milk in PBS with 0.1% Tween-20 (PBST) for one hour and probed overnight with anti-phosphotyrosine (05-321; Millipore), FGFR4, STAT3 (4904; Cell Signaling), and phospho-STAT3 (9131; Cell Signaling) antibodies. HRP-conjugated anti-mouse or anti-rabbit secondary antibodies (Thermo) were used to detect the primary antibodies. Finally, ECL (GE Biosciences) or SuperSignal (Thermo) was added and signal was detected on Biomax MR X-ray film (Kodak).

### *In vivo* Tumor Growth Assay

Animal studies were conducted with 6- to 8-week-old nude female, athymic NCr-nu/nu mice (Animal Production Program, SAIC-Frederick, MD). RMS772 transductants were used to assess *in vivo* tumor growth. Approximately 1 million cells were injected subcutaneously into the right flank of each mouse. Mice were monitored every other day. Tumor volume measurements were also performed every other day by caliper and the following formula was used to calculate tumor size: (long axis x short axis^2^)/2. Daily oral administration by gavage feeding of ponatinib at 30 mg/kg started when the tumor volume exceeded 100 mm^3^. Mice were euthanized when tumors reached 1,500 mm^3^.

### Oligonucleotides and Plasmids

pDonr253 is a Gateway Donor vector modified from pDonr201 (Life Technologies). pDonr253 replaces the kanamycin resistance gene with a gene encoding spectinomycin resistance, and contains several sequencing primer sites to aid in sequence verification of Entry clones. The following oligonucleotides (Eurofins MWG Operon) were used in this study:

7464: 5′- atgtctgagactcctgctcagtg

7465: 5′- ggagcggtgcaacagttcaatgg

7466: 5′- ccattgaactgttgcaccgctccCCCGCCTTGCTCGCCGGCCTCGTGAG

7467: 5′- tgtctgcaccccagacccgaagggg

7468: 5′- GGGGACAACTTTGTACAAAAAAGTTGGCACCATGtctgagactcctgctcagtg

7469: 5′- GGGGACAACTTTGTACAAGAAAGTTGATTAttatgtctgcaccccagacccgaagggg

### Cloning of TEL-FGFR4

The TEL-FGFR4 gene was constructed by fusing the kinase domain of FGFR4 in frame with the extracellular domain of TEL (Figure S1A).The TEL-FGFR4 chimera was cloned using overlap extension PCR from cDNA constructs for FGFR4 (Accession # BC011847) and ETV6/TEL (human ORFeome clone). Initial PCRs (left and right) were carried out using Phusion DNA polymerase (New England Biolabs) under standard conditions using a 30 second (TEL) or 60 second (FGFR4) extension time and 200 nM of flanking primers for 20 cycles. PCR products from these reactions were cleaned using the QiaQuick PCR purification kit (Qiagen), and equal amounts of each product pair were combined in a second 20 cycle PCR reaction using the flanking primers. These primers contain Gateway recombination signal sequences, attB1 at the 5′ end and attB2 at the 3′ end. The final PCR products were cleaned using the QiaQuick PCR purification kit (Qiagen), and recombined into pDonr253 using the Gateway BP recombination reaction (Life Technologies) per the manufacturer's protocols. BP reactions were transformed into E. coli DH10B cells, and colonies were isolated on LB plates containing 50 µg/mL spectinomycin. Plasmid DNA was prepared and sequenced using a variety of internal and external sequencing primers to verify the sequence.

### Subcloning of TEL-FGFR4 into a Retroviral Expression Vector

pMSCV-hyg (Clontech) was digested with HpaI and a Gateway reading frame cassette (Life Technologies) was introduced. Proper clones were selected for using ampicillin and chloramphenicol and sequence validated for directionality of the insert and proper sequence at the junctions. The modified Gateway Destination vector was called pDest-450 and was used for subcloning of the TEL-FGFR4 Entry clone via Gateway LR recombination (Life Technologies). Final clones were then transformed into E. coli STBL3 cells (Life Technologies) and final expression constructs were validated by agarose gel electrophoresis and restriction mapping.

### Retroviral Transfection of Ba/F3 Cells

Retrovirus containing the TEL-FGFR4 fusion gene was transfected into the PT67 packaging cells (Invitrogen) using Fugene 6 Transfection Reagent (Roche). Two days later, the cell culture supernatant containing the virus was collected, centrifuged, and filtered. Polybrene (Sigma-Aldrich) was added to the viral supernatant at a final concentration of 5 µg/mL. The resulting supernatant was then used to infect the Ba/F3 cells for eight hours. The stable, transfected cells were established by selecting cells in full media containing 1 mg/ml of hygromycin.

### RT-PCR of the PAX3/7-FOXO1 Fusion Gene

PAX3/7-FOXO1 fusion gene status was determined by RT-PCR. The same cDNA used in the quantitative RT-PCR was also used for this PCR (35 cycles of 95°C for 30 seconds, 60°C for 30 seconds, and 72°C for 45 seconds). We used the forward primer CCGACAGCAGCTCTGCCTAC and reverse primer ATGAACTTGCTGTGTAGGGACAG to amplify the fusion gene. PCR product was analyzed using the DNA 1000 kit (Agilent) to check for a 172 bp band.

### DNA Sequencing

We used the following PCR Primers for FGFR4 protein-coding exons 12 and 13—exon 12 forward: GATTCAGCCCTAGACCTACG; exon 12 reverse: CACTCCACGATCACGTAC; exon 13 forward: CAACCTGCTTGGTGTCTG; and exon 13 reverse: GGAAAGCGTGAATGCCTG. Cell line DNA was amplified by PCR and the product was confirmed by gel electrophoresis. PCR DNA was purified using the AMPure XP kit (Agencourt) and then sequenced by Sanger sequencing. Sequencing results were analyzed with Sequencher (Gene Codes).

## Results

### RMS cells with overexpressed wild-type FGFR4 are more sensitive to ponatinib

**Figure 1:**
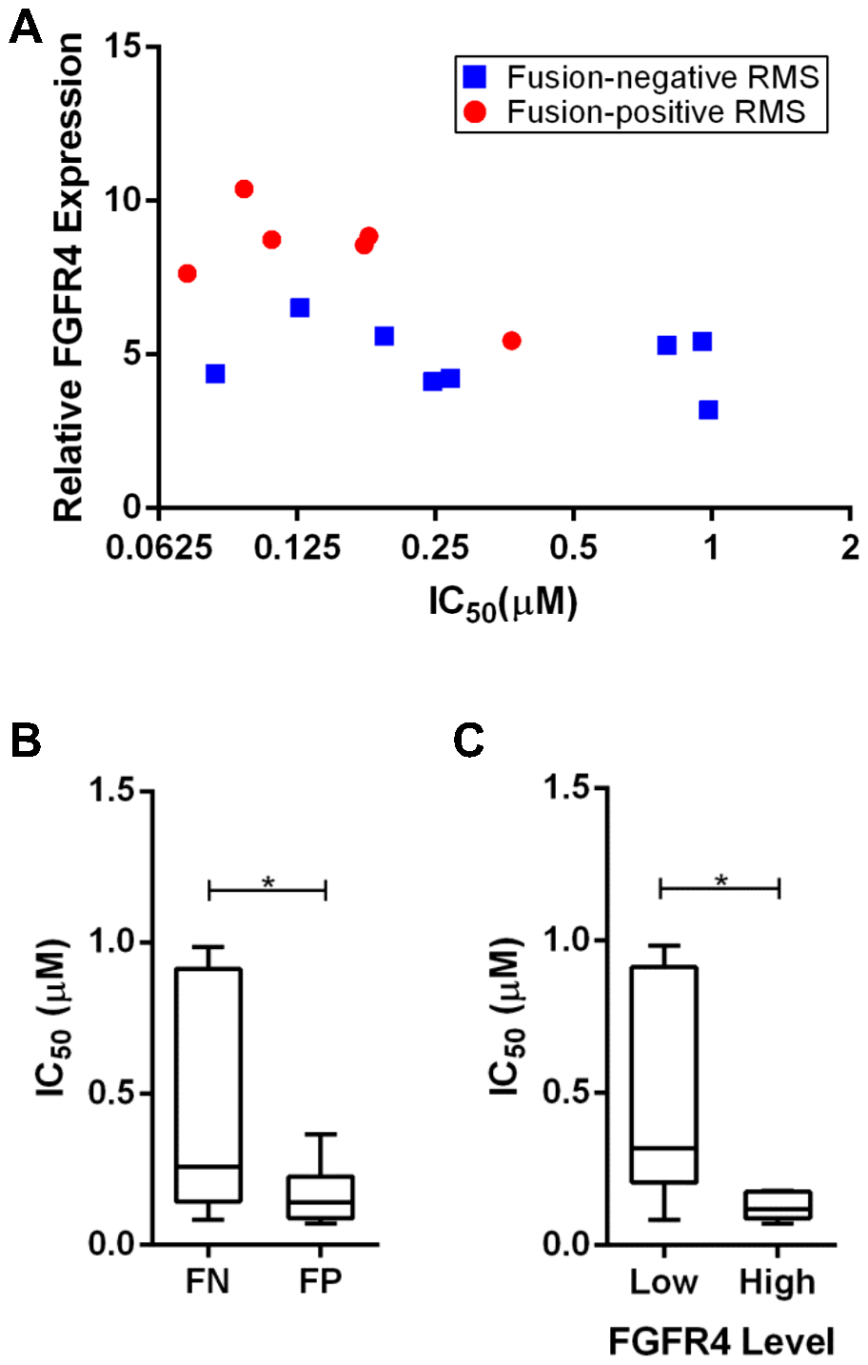
RMS cell lines with overexpressed FGFR4 are more sensitive to ponatinib. (A) The sensitivity of a panel of six fusion-positive RMS cell lines (RH4, RH28, JR, RH41, RH5, and RH30) and eight fusion-negative RMS cell lines (BIRCH, RH18, TTC-442, CT-10, CTR, TTC-516, RD, and RH36) to ponatinib is correlated to FGFR4 mRNA expression levels by Spearman ranking (p = 0.0261). (B) Comparing the variation in IC_50_ values of fusion-positive (FP) and fusion-negative (FN) RMS cell lines shows a significant difference by F test (p = 0.0125). (C) A difference in IC_50_ values can be seen between RMS cell lines expressing low (below a relative level of 6) and high (above a relative level of 6) levels of FGFR4 (p = 0.0344).

Using our Ba/F3 model system (Figure S1A-C), we confirmed previous reports that ponatinib is the most sensitive FGFR4 inhibitor with an IC_50_ of 72.2 nM (Figure S2A) and that it inhibits wild-type FGFR4 phosphorylation (Figure S2B) [16]. To demonstrate ponatinib's efficacy in RMS, we tested the molecule on a panel of RMS cell lines. We used six fusion-positive and eight fusion-negative RMS cell lines (fusion status verified by RT-PCR; Figure S3A). All tested cell lines showed nanomolar sensitivity to the drug and the sensitivity was dependent on the level of FGFR4 mRNA expression (Figure 1A, p = 0.0261, Spearman correlation). Of note, fusion-positive cell lines had significantly lower IC_50_s compared to fusion-negative cell lines, which have a wide range of sensitivity to ponatinib (Figure 1B, p = 0.0125, F-test). This is consistent with the fact that fusion-positive RMS cell lines typically express higher levels of FGFR4 (Figure S3B, p = 0.0005), because it is directly induced by the *PAX3-FOXO1* fusion gene [13]. Indeed, cell lines expressing the highest levels of FGFR4 (above a relative level of six) were the most sensitive to ponatinib (Figure 1C, p = 0.0344). These results, therefore, suggest that increased expression of FGFR4 confers sensitivity to ponatinib.

### RMS cells expressing activating FGFR4 mutations have increased sensitivity to ponatinib

**Figure 2:**
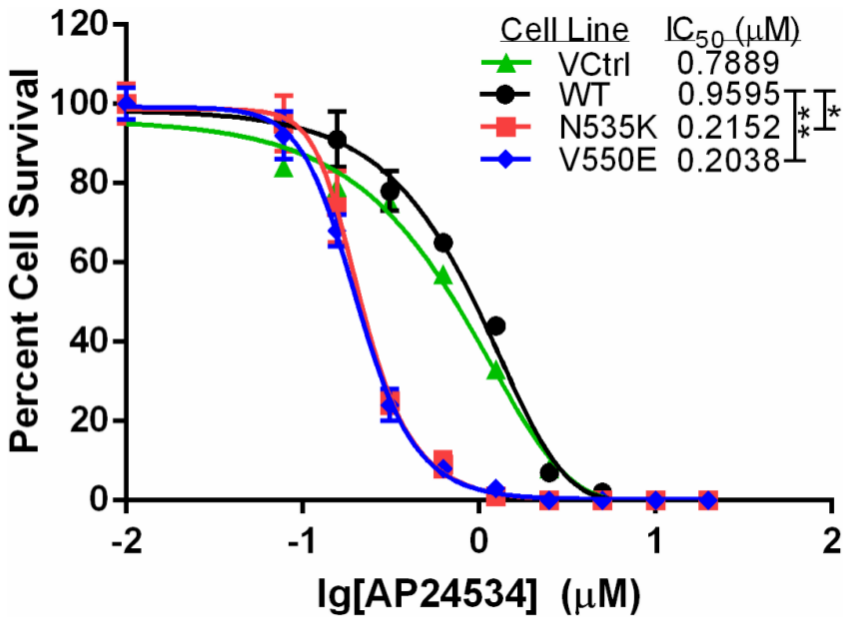
RMS772 cell harboring activating FGFR4 mutations V550E or N535K are more sensitive to ponatinib (AP24534) after 24 hour treatment than RMS772 cells expressing wild-type (WT) FGFR4 or the empty vector (VCtrl) (*p = <0.0001, **p = <0.0001).

The effect of ponatinib on RMS cells with mutationally activated FGFR4 was also tested in RMS772 cells engineered to express empty vector, wild-type FGFR4, FGFR4 N535K, and FGFR4 V550E [9], [17]. We found that the cells with the FGFR4 N535K and V550E mutation showed a significantly lower IC_50_ (215 and 204 nM, respectively) compared to wild-type FGFR4 (960 nM; Figure 2, *^,^**p<0.0001), suggesting that the activating FGFR4 mutations make cells more sensitive to ponatinib than wild-type FGFR4. Furthermore, we found in a kinetic study that the effect of ponatinib on cell confluency was rapid, within 6 hours, in RMS772 cell lines harboring the N535K or V550E mutations (Figure S4A). However, no significant difference in IC_50_ was seen between wild-type FGFR4 and the empty vector (Figure S4B).

### Ponatinib treatment results in decrease in S-phase fraction of cell cycle and augmented apoptosis in RMS cells

**Figure 3:**
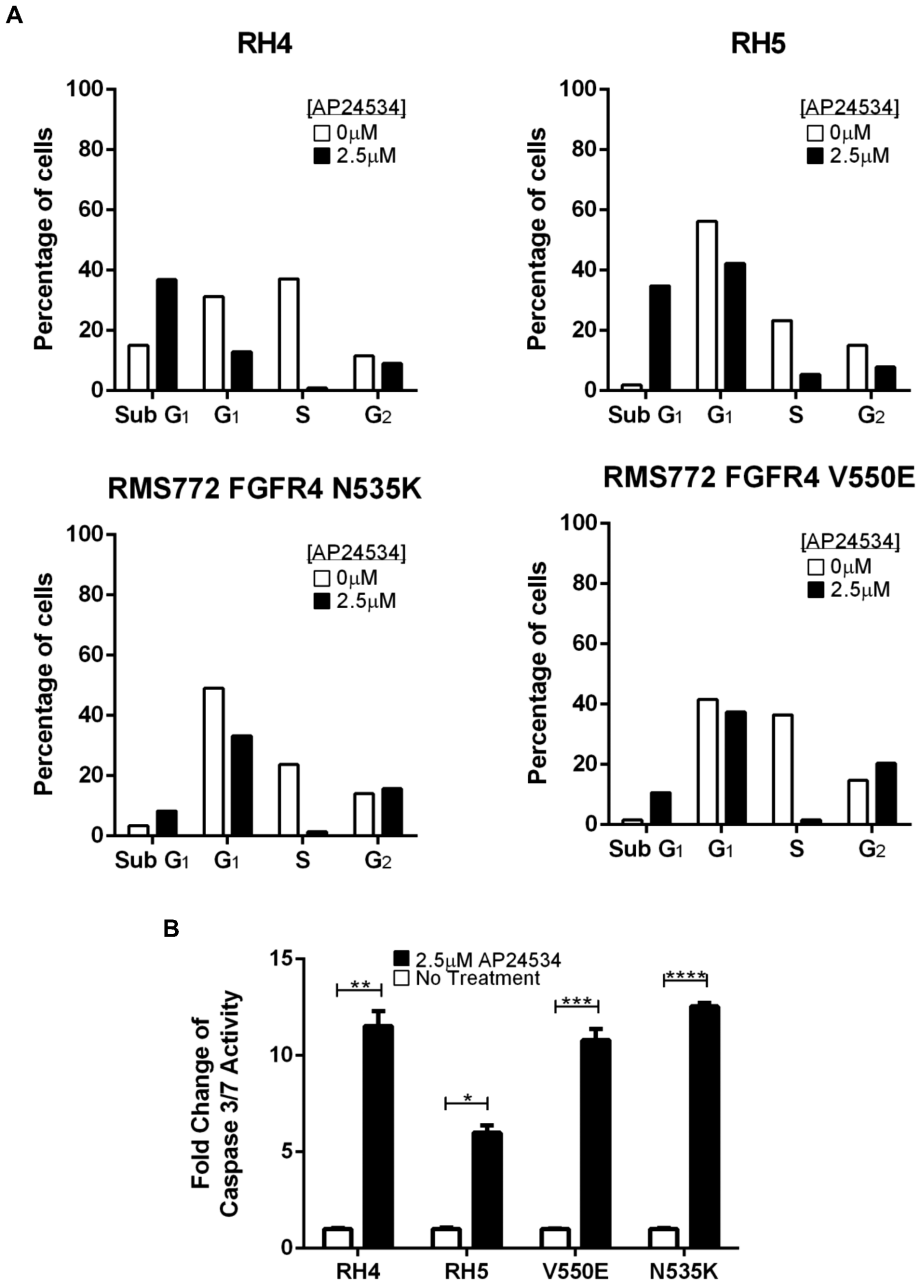
Ponatinib (AP24534) holds cell cycling at sub G_1_ phase and induces cell death via apoptosis. (A) Cell cycle analysis of the two most sensitive cell lines to ponatinib, RH4 and RH5, and the two RMS772 cell lines expressing FGFR4 mutations (N535K and V550E) showed increased time in sub G_1_ phase and decreased time in S phase across all four cell lines after 24 hours of treatment with 2.5 µM ponatinib. (B) Cell death induced by 2.5 µM ponatinib treatment is mediated *via* the caspase 3/7 pathway (*p = 0.0029, **p = 0.0027, ***p = 0.0017, ****p = 0.0001).

To investigate the effects of ponatinib on cell cycling, we tested two RMS cell lines, RH4 and RH5, which displayed the highest sensitivity to ponatinib and the two RMS772 cell lines which express mutationally activated FGFR4s. After three hour exposure to ponatinib at final concentrations of 0, 0.625, 1.25, and 2.5 µM, BrdU incorporation showed a decrease in S phase and an increase in sub G_1_ across all four cell lines, indicating reduced cell growth and increased apoptosis, respectively (Figure 3A, Figures S5 and S6). Furthermore, a caspase 3/7 assay demonstrated that similar concentrations of ponatinib induced apoptosis in all four cell lines (Figure 3B, *p = 0.0029, **p = 0.0027, ***p = 0.0017, ****p = 0.0001; Figure S7).

### Ponatinib inhibits the phosphorylation of wild-type and mutationally activated FGFR4 and its downstream target STAT3 in a dose-dependent manner

**Figure 4:**
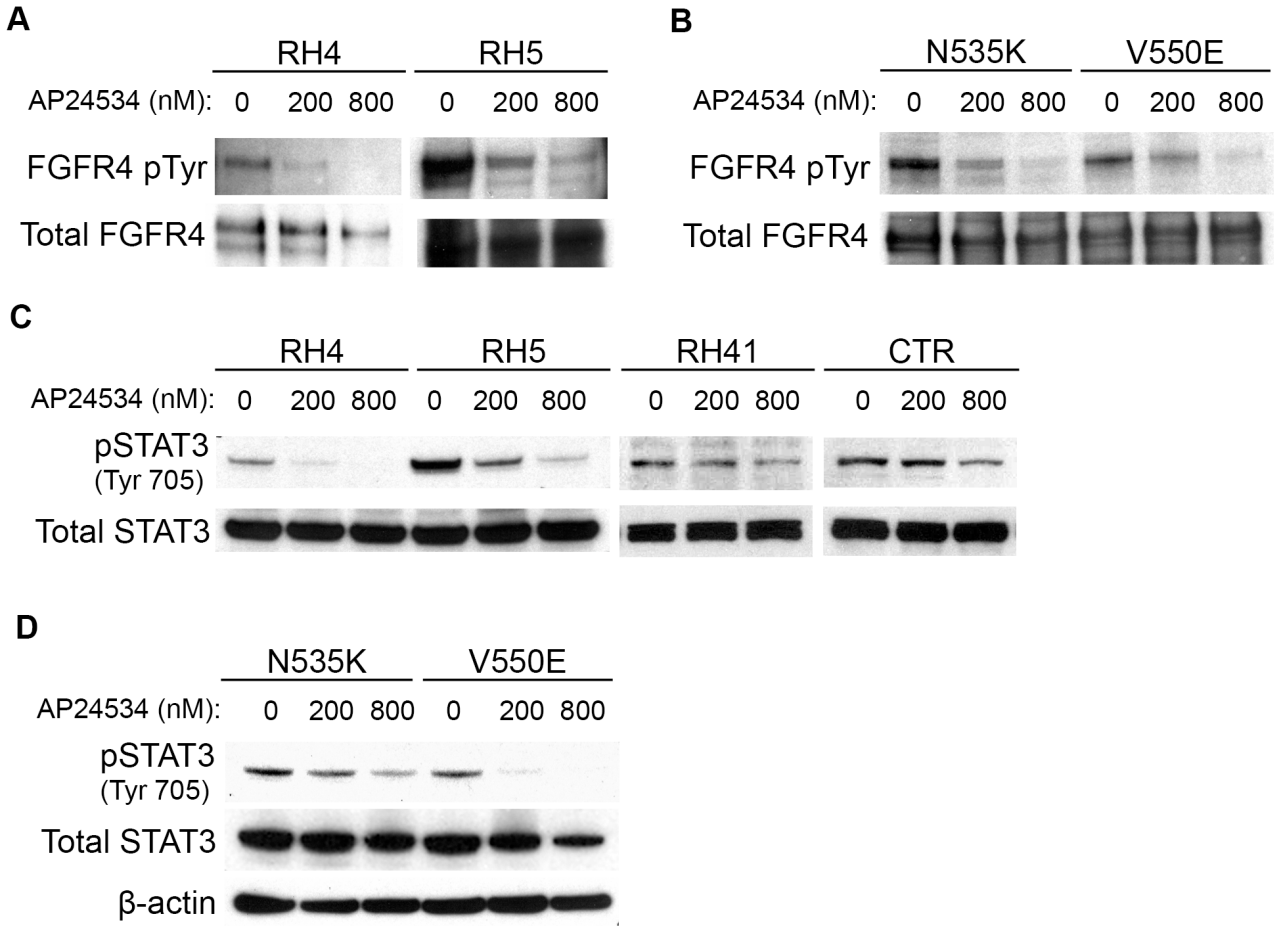
Western blot analysis of expression and phosphorylation of wild-type and mutated FGFR4 and its downstream target, STAT3, after treatment with 0, 200, and 800 nM concentrations of ponatinib (AP24534) for 8 hours. (A) A dose-dependent decrease in wild-type FGFR4 phosphorylation as shown by immunoprecipitation of FGFR4 and immunoblotting for phosphotyrosine. (B) A similar dose-dependent inhibition is seen for FGFR4 with the V550E and N535K mutation. (C-D) Western blot shows a dose-dependent decrease in STAT3 phosphorylation after treatment with ponatinib for three fusion-positive (RH4, RH5, and RH41) and one fusion-negative (CTR) RMS cell lines as well as the two RMS772 cell lines expressing the FGFR4 mutations N535K and V550E.

To determine whether ponatinib inhibits the phosphorylation of wild-type and mutationally activated FGFR4, we performed western blot analysis on cells treated with ponatinib for eight hours. Wild-type FGFR4 phosphorylation was inhibited by ponatinib in a dose-dependent manner for two fusion-positive cell lines, RH4 and RH5 (Figure 4A), as well as the mutationally activated FGFR4 cell lines, RMS772/FGFR4 N535K and FGFR4 V550E (Figure 4B).

We have previously reported that STAT3 is a downstream target of FGFR4 [9]. Therefore, we investigated the effect of ponatinib on the phosphorylation of STAT3 and found a dose-dependent reduction of STAT3 phosphorylation in three fusion-positive (RH4, RH5, and RH41) and a fusion-negative cell lines (CTR; Figure 4C), as well as the mutationally activated FGFR4 cell lines (Figure 4D).

### Ponatinib inhibits mutant FGFR4-driven RMS tumor growth *in vivo*

**Figure 5:**
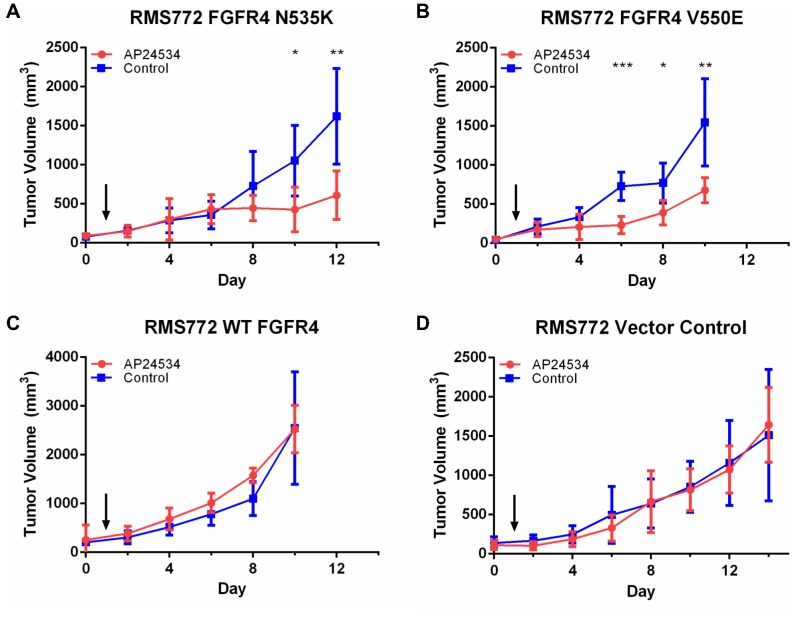
*In vivo* tumor growth assay with daily treatment of 30 mg/kg of ponatinib after tumor volumes reach 100 mm^3^. Arrow indicates the start of treatment. (A) Treatment of tumors harboring the FGFR4 N535K mutation with ponatinib significantly inhibits tumor growth after 10 days of treatment (*p = 0.0165, **p = 0.0048). (B) Treatment of tumors containing the FGFR4 V550E mutation with ponatinib significantly inhibits tumor growth after 6 days of treatment (*p = 0.0185, **p = 0.0087, ***p = 0.0005). (C) Treatment of tumors expressing the wild-type FGFR4 with ponatinib does not affect tumor growth. (D) Treatment of tumors expressing the empty vector with ponatinib does not affect tumor growth.

We then used a previously reported mouse xenograft model [9] to test the efficacy of ponatinib *in vivo* against RMS cells harboring the constitutively activating FGFR4 mutations. Mice were injected subcutaneously with the RMS772 cell lines stably expressing the empty vector, FGFR4 WT, FGFR4 N535K, or FGFR4 V550E. After 10 days of treatment, the tumor sizes of mice bearing the two mutant FGFR4 RMS772 cell lines were significantly smaller compared to their untreated counterpart (Figure 5A and 5B). However, there was no difference in tumor volume for mice injected with the RMS772 FGFR4 WT or empty vector cell line when treated with or without ponatinib (Figure 5C and 5D), indicating that RMS tumors with activating FGFR4 mutations at their tyrosine kinase domain may be more sensitive to the inhibition of ponatinib *in vivo*.

## Discussion

Alteration of FGFR4 signaling is a common mechanism of oncogenesis in both fusion positive and fusion negative rhabdomyosarcoma (RMS). Thus far, at least three mechanisms have been reported to result in the gain of function of FGFR4 in RMS. First, elevated FGFR4 expression in RMS tumors can be a direct result of the *PAX3-FOXO1* fusion oncogene, since FGFR4 was reported to be one of the direct targets of the transcription factor [13]. Secondly, up-regulation of FGFR4 expression in RMS can be achieved through localized gene amplification [22]. Thirdly, 7.5% of primary RMS tumors harbor a damaging missense mutation in the tyrosine kinase domain of FGFR4 which results in a constitutively active signaling molecule [9]. The first two mechanisms result in elevated expression of wild-type FGFR4 in RMS, which is both common and associated with poor outcome [9], [12], [23]. Previous studies have also shown that knockdown of FGFR4 in RMS cell lines results in inhibition of cell proliferation *in vitro* and metastasis *in vivo* [9], [24]. The third mechanism of somatic mutation of FGFR4 results in the constitutive, ligand-independent activation of FGFR4 [25]. Given these findings, we hypothesized that inhibition of FGFR4 signaling would be an effective strategy for the treatment of RMS.

In our previous study, we have shown that RMS772 cell lines expressing the FGFR4 V550E or N535K mutation were sensitive to PD173074, a FGFR inhibitor, in the micromolar range [9]. To find FGFR inhibitors that are more effective, we searched for small molecule tyrosine kinase inhibitors that inhibit a FGFR member in clinically achievable concentrations and found five candidate compounds: AP24534 (ponatinib), AZD2171 (Cediranib), BIBF1120 (Vargatef), TKI258 (Dovitunib), and PHA739358 (Danusertib) (Table S1). It is noteworthy to mention that ponatinib has received accelerated FDA approval for treatment of adult patients with Ph+ ALL or chronic phase, accelerated phase, or blast phase CML who are resistant or intolerant to prior tyrosine kinase inhibitor therapy. Using Ba/F3 cells, we found that transduction of TEL-FGFR4 results in activation of FGFR4 via autophosphorylation and IL-3 independent survival and growth (Figure S1A-C). Furthermore, we screened the five FGFR inhibitors with this Ba/F3 TEL-FGFR4 model and verified that ponatinib is the most potent FGFR4 inhibitor among those tested [16].

Consistent with these findings, ponatinib inhibited the growth of multiple fusion-positive and fusion-negative RMS cell lines, all with IC_50_ values in the nanomolar range. Our data confirmed that FGFR4 mRNA expression was significantly higher in fusion-positive cell lines than fusion-negative cell lines (Figure S3B, p = 0.0005) [9], [24]. In addition, the sensitivity to ponatinib correlated with FGFR4 mRNA expression levels and that fusion-positive cell lines with the higher FGFR4-expressing levels were consistently sensitive to ponatinib. However, cell lines that were fusion-negative or expressed FGFR4 at low levels (less than a relative level of 6) had a wider variation of ponatinib sensitivity. Therefore, it is possible that a certain threshold of FGFR4 expression is needed for consistent nanomolar sensitivity to ponatinib. In addition, no mutations were found in any of the cell lines by Sanger sequencing of exon 12 and 13 of FGFR4 (data not shown), eliminating the possibility of a mutation conferring sensitivity in any of the studied cell lines. However, since ponatinib is a multikinase inhibitor, which includes inhibition of RET, LYN, LCK, FYN, and ABL at subnanomolar concentrations (Table S2), it is possible that the activity is related to the inhibition of other kinases. Indeed, even some cell lines with lower levels of FGFR4 expression continue to demonstrate sensitivity to ponatinib and it is possible that this effect may be the result of inhibition of targets other than FGFR4. This is demonstrated in normal skin fibroblast, osteosarcoma, and Ewing's sarcoma cell lines (Figure S8).

Similar to the RMS cell lines with high expression of wild-type FGFR4, we have shown ponatinib to be effective against a RMS model system with constitutively activating FGFR4 mutations N535K and V550E. After treatment of cells expressing the mutated FGFR4 with ponatinib, IC_50_ values were achieved in the nanomolar range within 24 hours (Figure S4A-B). We found there was G_1_/S arrest of cell cycling with an increase in the sub G_1_ phase fraction indicating cell death, which was confirmed by caspase 3/7 induction.

It is interesting to note that the *in vitro* data shows ponatinib to be effective against wild-type and mutant FGFR4, whereas our *in vivo* results show that ponatinib only inhibits tumor growth of cells harboring the FGFR4 mutations but not the wild-type FGFR4. One possible reason for this may come from our observation that the murine RMS cells expressing wild-type FGFR4 have a higher IC_50_ than the cells expressing the two mutant FGFR4s. Therefore a higher inhibitory dosage than what was used may be necessary for the treatment of wild-type FGFR4 in order to observe an effect on tumor xenograft growth. Another possible reason for this may be due to the model system we use: our murine RMS772 cell line which artificially expresses human wild-type FGFR4. Although this models human embryonal rhabdomyosarcoma most closely, expressing human wild-type FGFR4 in a mouse cell or growing in an environment with murine stromal growth factors may alter its behavior differently. For example, we have previously shown that human wild-type FGFR4 does not increase growth or migration like mutated FGFR4 does in RMS772 cells [9]. Given our findings, we believe that targeting FGFR4 will be most effective in ERMS with high expression (due to amplification) or mutation of FGFR4 or in alveolar rhabdomyosarcoma (ARMS) where the PAX3/7-FOXO1 fusion gene found in ARMS directly increases expression of FGFR4. Future studies regarding this observation are being actively pursued using *in vivo* studies of human rhabdomyosarcoma cell lines.

Biochemically, we found that ponatinib effectively decreased phosphorylation of wild type and mutant FGFR4 in a dose-dependent manner, indicating that at least one of its biological effects is through targeting FGFR4 kinase function. We also investigated STAT3 phosphorylation because the expression level of this gene is known to be high in RMS [12] and we have previously shown it to be activated by the FGFR4 mutations V550E and N535K as a downstream target of FGFR4 [9]. We found that STAT3 phosphorylation was also inhibited by ponatinib in a dose-dependent manner. Further dissection of the underlying molecular mechanism is underway to determine whether STAT3 is inhibited by ponatinib directly or via the FGFR4 pathway.

In summary, we find ponatinib as a multi-targeted tyrosine kinase inhibitor that displays potent pan-FGFR activity including nanomolar inhibition of FGFR4. In our FGFR inhibitor screen, ponatinib was identified as the most potent FGFR4 inhibitor, successfully inhibiting FGFR4 and its downstream STAT3 phosphorylation. Furthermore, ponatinib caused G_1_/S arrest of cell cycling and inhibition of *in vivo* tumor growth of RMS cells expressing the FGFR4 N535K and V550E mutations. Given that this molecule has demonstrated an acceptable efficacy and safety profile in ongoing clinical trials and that there is an urgent need to develop novel therapies for patients suffering from RMS, our presented pre-clinical findings strongly support further clinical investigation of ponatinib for RMS patients with overexpressed or mutationally activated FGFR4.

## Supporting Information

Figure S1 **The TEL-FGFR4 model system in Ba/F3 cells.** (A) The TEL-FGFR4 construct was created by fusing the extracellular PNT domain of ETV6/TEL in frame with the intracellular kinase domain of FGFR4. ETV6/TEL consists of a pointed (PNT) domain, which polymerizes, and an ETS domain that binds to DNA. FGFR4 contains three extracellular immunoglobulin (IG) domains, a transmembrane domain (unlabeled), and an intercellular tyrosine kinase (TK) domain. Numbers represent start and end sites of domains along the amino acid sequence. (B) Expression and autophosphorylation of FGFR4 is present in the Ba/F3 TEL-FGFR4 model system as shown by immunoprecipitation of FGFR4 and then western blotting against phosphotyrosine. Expression and autophosphorylation of FGFR4 is not present in Ba/F3 cells expressing the empty vector. β-actin was probed as well to ensure equal loading of protein. (C) Ba/F3 cells that were retrovirally transfected with the TEL-FGFR4 construct survived independently of IL-3 over 72 hours. However, Ba/F3 cells expressing the vector control only survived with IL-3 supplementation.
(PPTX)

Figure S2 **Ponatinib is the most potent FGFR4 inhibitor and inhibits wild-type FGFR4 phosphorylation.** (A) FGFR inhibitor screen with the Ba/F3 TEL-FGFR4 model system shows ponatinib (AP24534) to be the most potent inhibitor among the four other FGFR inhibitors, TKI258, BIBF1120, PHA739358, and AZD2172, in addition to the MET inhibitor, PHA665752, as a control. (B) Immunoprecipitation of FGFR4 and immunoblotting against phosphotyrosine shows a dose-dependent inhibition of FGFR4 phosphorylation with ponatinib using the Ba/F3 TEL-FGFR4 model system.
(PPTX)

Figure S3 **PAX3/7-FOXO1 fusion status of cell lines and fusion-positive RMS cell lines express higher levels of FGFR4 mRNA.** (A) RT-PCR with a PAX3/7-FOXO1 primer (forward: CCGACAGCAGCTCTGCCTAC and reverse: ATGAACTTGCTGTGTAGGGACAG) shows cell lines RH5, RH4, JR, RH41, RH28, and RH30 to be fusion-positive while cell lines RH18, CTR, BIRCH, RD, TTC-516, CT-10, TTC-442, and RH36 to be fusion-negative. The PAX3/7-FOXO1 band appears at 172 bp. (B) Comparison of FGFR4 mRNA expression levels between fusion-positive (FP) and fusion-negative (FN) cell lines reveals fusion-positive cell lines to express FGFR4 at higher levels than fusion-negative cell lines (p = 0.0005). FGFR4 expression was normalized to GAPDH expression.
(PPTX)

Figure S4 **Kinetic analysis of ponatinib-induced growth inhibition for the RMS772 transductants as measured by confluency.** (A) Growth curves for the RMS772 transductants illustrate differential sensitivity to ponatinib. Arrow indicates when ponatinib was added. (B) IC_50_ calculation at 6, 12, and 24 hours after the addition of ponatinib for RMS772 transductants shows a decrease in IC_50_ as time increases.
(PPTX)

Figure S5 **Ponatinib (AP24534) holds cell cycling at sub G_1_ phase and decreases time in S phase when RH4, RH5, and the two RMS772 cell lines expressing FGFR4 mutations (N535K and V550E) are treated with 0.625 and 1.25 µM ponatinib for 24 hours.**
(PPTX)

Figure S6 **Dot plot of cell cycle flow cytometry data showing the boxes used to determine cell cycle fraction for** **Figure 3A** **and Figure S5.** Each box represents a different cell cycle phase, starting with the top box and going clockwise: S phase, G_2_ phase, G_1_ phase, and subG_1_ phase.
(PPTX)

Figure S7 **Treatment of cell lines RH4, RH5, and the two RMS772 cell lines expressing mutated FGFR4 with 0, 1.25, 2.5, and 5 µM ponatinib for 6 hours increases caspase 3/7 levels across all four cell lines.**
(PPTX)

Figure S8 **Fitted drug-dose response curves and calculated IC_50_s for cell lines (A) 7250 LNCX NILC, (B) U2-OS, and (C) A4573 after 72 hour treatment with ponatinib.**
(PPTX)

Table S1 FGFR inhibition profiles for tested inhibitors.
(XLSX)

Table S2 Kinase inhibition profile of tested FGFR inhibitors [14], [16], [26]–[32].
(XLSX)

## References

[1] Ognjanovic S, Linabery AM, Charbonneau B, Ross JA (2009) Trends in Childhood Rhabdomyosarcoma Incidence and Survival in the United States, 1975–2005. Cancer 115: 4218–4226. 19536876 10.1002/cncr.24465 PMC2953716

[2] Malempati S, Hawkins DS (2012) Rhabdomyosarcoma: Review of the Children's Oncology Group (COG) soft-tissue Sarcoma committee experience and rationale for current COG studies. Pediatric Blood & Cancer 59: 5–10. 22378628 10.1002/pbc.24118 PMC4008325

[3] Breneman JC, Lyden E, Pappo AS, Link MP, Anderson JR, et al (2003) Prognostic factors and clinical outcomes in children and adolescents with metastatic rhabdomyosarcoma--a report from the Intergroup Rhabdomyosarcoma Study IV. J Clin Oncol 21: 78–84. 12506174 10.1200/JCO.2003.06.129

[4] Barr FG, Galili N, Holick J, Biegel JA, Rovera G, et al (1993) Rearrangement of the PAX3 paired box gene in the paediatric solid tumour alveolar rhabdomyosarcoma. Nat Genet 3: 113–117. 8098985 10.1038/ng0293-113

[5] Scrable H, Cavenee W, Ghavimi F, Lovell M, Morgan K, et al (1989) A model for embryonal rhabdomyosarcoma tumorigenesis that involves genome imprinting. Proc Natl Acad Sci U S A 86: 7480–7484. 2798419 10.1073/pnas.86.19.7480 PMC298088

[6] Taylor AC, Shu L, Danks MK, Poquette CA, Shetty S, et al (2000) P53 mutation and MDM2 amplification frequency in pediatric rhabdomyosarcoma tumors and cell lines. Med Pediatr Oncol 35: 96–103. 10918230 10.1002/1096-911x(200008)35:2<96::aid-mpo2>3.0.co;2-z

[7] Stratton MR, Fisher C, Gusterson BA, Cooper CS (1989) Detection of point mutations in N-ras and K-ras genes of human embryonal rhabdomyosarcomas using oligonucleotide probes and the polymerase chain reaction. Cancer Res 49: 6324–6327. 2680062

[8] Shukla N, Ameur N, Yilmaz I, Nafa K, Lau CY, et al (2012) Oncogene mutation profiling of pediatric solid tumors reveals significant subsets of embryonal rhabdomyosarcoma and neuroblastoma with mutated genes in growth signaling pathways. Clin Cancer Res 18: 748–757. 22142829 10.1158/1078-0432.CCR-11-2056 PMC3271129

[9] Taylor JGt, Cheuk AT, Tsang PS, Chung JY, Song YK, et al (2009) Identification of FGFR4-activating mutations in human rhabdomyosarcomas that promote metastasis in xenotransplanted models. J Clin Invest 119: 3395–3407. 19809159 10.1172/JCI39703 PMC2769177

[10] Zhao P, Caretti G, Mitchell S, McKeehan WL, Boskey AL, et al (2006) Fgfr4 is required for effective muscle regeneration in vivo - Delineation of a MyoD-Tead2-Fgfr4 transcriptional pathway. Journal of Biological Chemistry 281: 429–438. 16267055 10.1074/jbc.M507440200 PMC1892582

[11] Marics I, Padilla F, Guillemot JF, Scaal M, Marcelle C (2002) FGFR4 signaling is a necessary step in limb muscle differentiation. Development 129: 4559–4569. 12223412 10.1242/dev.129.19.4559

[12] Khan J, Wei JS, Ringner M, Saal LH, Ladanyi M, et al (2001) Classification and diagnostic prediction of cancers using gene expression profiling and artificial neural networks. Nature Medicine 7: 673–679. 10.1038/89044 PMC1282521 11385503

[13] Cao L, Yu Y, Bilke S, Walker RL, Mayeenuddin LH, et al. Genome-wide identification of PAX3-FKHR binding sites in rhabdomyosarcoma reveals candidate target genes important for development and cancer. Cancer Res 70: 6497–6508. 20663909 10.1158/0008-5472.CAN-10-0582 PMC2922412

[14] O'Hare T, Shakespeare WC, Zhu XT, Eide CA, Rivera VM, et al (2009) AP24534, a Pan-BCR-ABL Inhibitor for Chronic Myeloid Leukemia, Potently Inhibits the T315I Mutant and Overcomes Mutation-Based Resistance. Cancer Cell 16: 401–412. 19878872 10.1016/j.ccr.2009.09.028 PMC2804470

[15] Gozgit JM, Wong MJ, Wardwell S, Tyner JW, Loriaux MM, et al (2011) Potent Activity of Ponatinib (AP24534) in Models of FLT3-Driven Acute Myeloid Leukemia and Other Hematologic Malignancies. Molecular Cancer Therapeutics 10: 1028–1035. 21482694 10.1158/1535-7163.MCT-10-1044 PMC3236248

[16] Gozgit JM, Wong MJ, Moran L, Wardwell S, Mohemmad QK, et al (2012) Ponatinib (AP24534), a multitargeted pan-FGFR inhibitor with activity in multiple FGFR-amplified or mutated cancer models. Mol Cancer Ther 11: 690–699. 22238366 10.1158/1535-7163.MCT-11-0450

[17] Yu Y, Khan J, Khanna C, Helman L, Meltzer PS, et al (2004) Expression profiling identifies the cytoskeletal organizer ezrin and the developmental homeoprotein Six-1 as key metastatic regulators. Nature Medicine 10: 175–181. 10.1038/nm966 14704789

[18] Hu K, Lee C, Qiu D, Fotovati A, Davies A, et al (2009) Small interfering RNA library screen of human kinases and phosphatases identifies polo-like kinase 1 as a promising new target for the treatment of pediatric rhabdomyosarcomas. Mol Cancer Ther 8: 3024–3035. 19887553 10.1158/1535-7163.MCT-09-0365 PMC2783569

[19] Thuault S, Hayashi S, Lagirand-Cantaloube J, Plutoni C, Comunale F, et al.. (2012) P-cadherin is a direct PAX3-FOXO1A target involved in alveolar rhabdomyosarcoma aggressiveness. Oncogene. 10.1038/onc.2012.217 22710718

[20] Solomon DA, Kim T, Diaz-Martinez LA, Fair J, Elkahloun AG, et al (2011) Mutational inactivation of STAG2 causes aneuploidy in human cancer. Science 333: 1039–1043. 21852505 10.1126/science.1203619 PMC3374335

[21] Stauffer JK, Orentas RJ, Lincoln E, Khan T, Salcedo R, et al (2012) High-throughput molecular and histopathologic profiling of tumor tissue in a novel transplantable model of murine neuroblastoma: new tools for pediatric drug discovery. Cancer Invest 30: 343–363. 22571338 10.3109/07357907.2012.664670 PMC6993178

[22] Paulson V, Chandler G, Rakheja D, Galindo RL, Wilson K, et al (2011) High-resolution array CGH identifies common mechanisms that drive embryonal rhabdomyosarcoma pathogenesis. Genes Chromosomes Cancer 50: 397–408. 21412928 10.1002/gcc.20864

[23] Davicioni E, Finckenstein FG, Shahbazian V, Buckley JD, Triche TJ, et al (2006) Identification of a PAX-FKHR gene expression signature that defines molecular classes and determines the prognosis of alveolar rhabdomyosarcomas. Cancer Res 66: 6936–6946. 16849537 10.1158/0008-5472.CAN-05-4578

[24] Crose LE, Etheridge KT, Chen C, Belyea B, Talbot LJ, et al (2012) FGFR4 blockade exerts distinct antitumorigenic effects in human embryonal versus alveolar rhabdomyosarcoma. Clin Cancer Res 18: 3780–3790. 22648271 10.1158/1078-0432.CCR-10-3063 PMC3713717

[25] Klint P, Claesson-Welsh L (1999) Signal transduction by fibroblast growth factor receptors. Front Biosci 4: D165–177. 9989949 10.2741/klint

[26] Wedge SR, Kendrew J, Hennequin LF, Valentine PJ, Barry ST, et al (2005) AZD2171: a highly potent, orally bioavailable, vascular endothelial growth factor receptor-2 tyrosine kinase inhibitor for the treatment of cancer. Cancer Res 65: 4389–4400. 15899831 10.1158/0008-5472.CAN-04-4409

[27] Hilberg F, Roth GJ, Krssak M, Kautschitsch S, Sommergruber W, et al (2008) BIBF 1120: triple angiokinase inhibitor with sustained receptor blockade and good antitumor efficacy. Cancer Res 68: 4774–4782. 18559524 10.1158/0008-5472.CAN-07-6307

[28] Trudel S, Li ZH, Wei E, Wiesmann M, Chang H, et al (2005) CHIR-258, a novel, multitargeted tyrosine kinase inhibitor for the potential treatment of t(4;14) multiple myeloma. Blood 105: 2941–2948. 15598814 10.1182/blood-2004-10-3913

[29] Lee SH, Lopes de Menezes D, Vora J, Harris A, Ye H, et al (2005) In vivo target modulation and biological activity of CHIR-258, a multitargeted growth factor receptor kinase inhibitor, in colon cancer models. Clin Cancer Res 11: 3633–3641. 15897558 10.1158/1078-0432.CCR-04-2129

[30] Carpinelli P, Ceruti R, Giorgini ML, Cappella P, Gianellini L, et al (2007) PHA-739358, a potent inhibitor of Aurora kinases with a selective target inhibition profile relevant to cancer. Mol Cancer Ther 6: 3158–3168. 18089710 10.1158/1535-7163.MCT-07-0444

[31] Fancelli D, Moll J, Varasi M, Bravo R, Artico R, et al (2006) 1,4,5,6-tetrahydropyrrolo[3,4-c]pyrazoles: identification of a potent Aurora kinase inhibitor with a favorable antitumor kinase inhibition profile. J Med Chem 49: 7247–7251. 17125279 10.1021/jm060897w

[32] Christensen JG, Schreck R, Burrows J, Kuruganti P, Chan E, et al (2003) A selective small molecule inhibitor of c-Met kinase inhibits c-Met-dependent phenotypes in vitro and exhibits cytoreductive antitumor activity in vivo. Cancer Res 63: 7345–7355. 14612533

