# Supplementary material for: Targeting Wild-Type and Mutationally Activated FGFR4 in Rhabdomyosarcoma with the Inhibitor Ponatinib (AP24534)
Source: PLoS One. 2013 Oct 4;8(10):e76551. doi: 10.1371/journal.pone.0076551 (PMC3790700; doi:10.1371/journal.pone.0076551)
Supplement: Figure S1 — The TEL-FGFR4 model system in Ba/F3 cells. (A) The TEL-FGFR4 construct was created by fusing the extracellular PNT domain of ETV6/TEL in frame with the intracellular kinase domain of FGFR4. ETV6/TEL consists of a pointed (PNT) domain, which polymerizes, and an ETS domain that binds to DNA. FGFR4 contains three extracellular immunoglobulin (IG) domains, a transmembrane domain (unlabeled), and an intercellular tyrosine kinase (TK) domain. Numbers represent start and end sites of domains along the amino acid sequence. (B) Expression and autophosphorylation of FGFR4 is present in the Ba/F3 TEL-FGFR4 model system as shown by immunoprecipitation of FGFR4 and then western blotting against phosphotyrosine. Expression and autophosphorylation of FGFR4 is not present in Ba/F3 cells expressing the empty vector. β-actin was probed as well to ensure equal loading of protein. (C) Ba/F3 cells that were retrovirally transfected with the TEL-FGFR4 construct survived independently of IL-3 over 72 hours. However, Ba/F3 cells expressing the vector control only survived with IL-3 supplementation. (PPTX) [file pone.0076551.s001.pptx]

## Slide 1
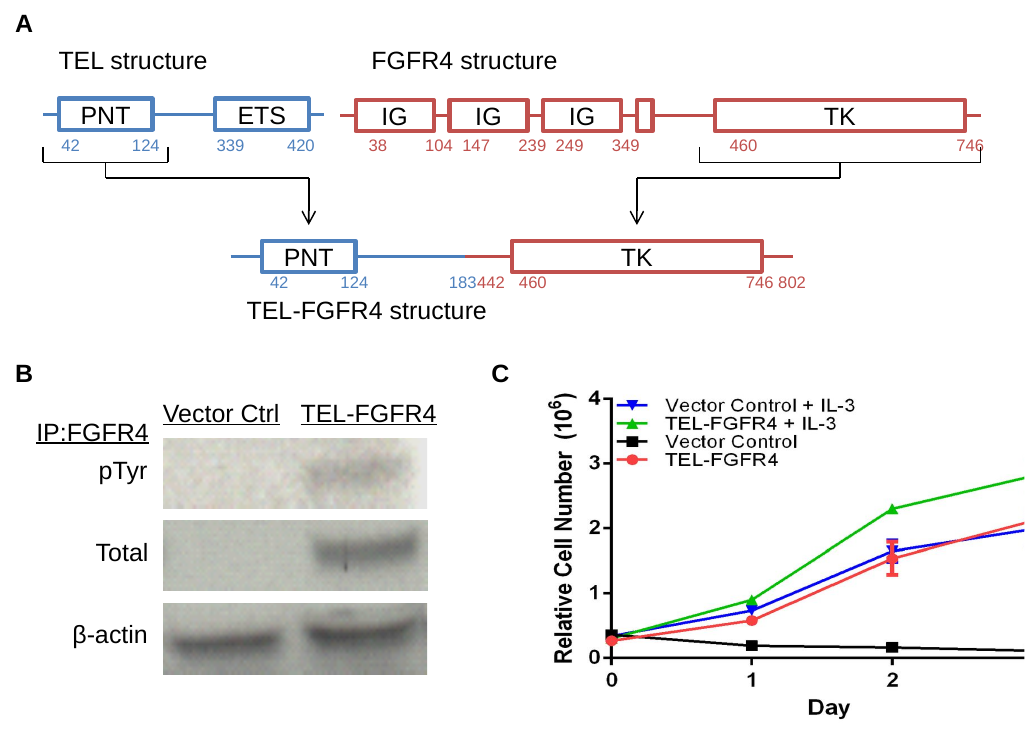

A
TEL structure
FGFR4 structure
PNT
ETS
IG
IG
IG
TK
42 124 339 420
38 104 147 239 249 349 460 746
PNT
TK
42 124 183442 460 746 802
TEL-FGFR4 structure
B
C
Vector Ctrl TEL-FGFR4
IP:FGFR4
pTyr
Total
β-actin
