# Supplementary figures and images for: Targeting Wild-Type and Mutationally Activated FGFR4 in Rhabdomyosarcoma with the Inhibitor Ponatinib (AP24534)
Source: PLoS One. 2013 Oct 4;8(10):e76551. doi: 10.1371/journal.pone.0076551 (PMC3790700; doi:10.1371/journal.pone.0076551)

## Slide 1
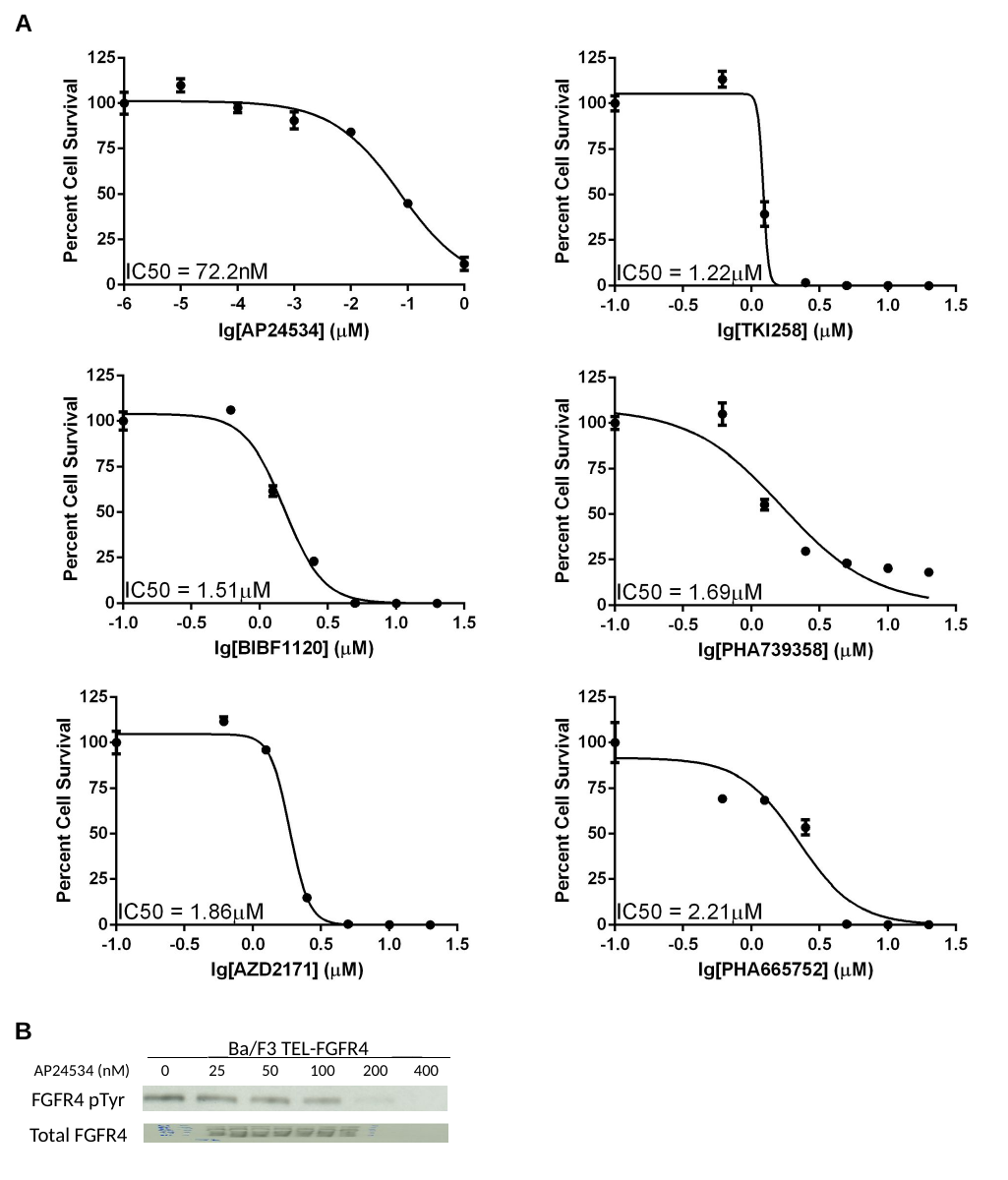

A
B
 __Ba/F3 TEL-FGFR4 ___
AP24534 (nM)
25
50
0
100
200
400
FGFR4 pTyr
Total FGFR4

Supplement: Figure S2 — Ponatinib is the most potent FGFR4 inhibitor and inhibits wild-type FGFR4 phosphorylation. (A) FGFR inhibitor screen with the Ba/F3 TEL-FGFR4 model system shows ponatinib (AP24534) to be the most potent inhibitor among the four other FGFR inhibitors, TKI258, BIBF1120, PHA739358, and AZD2172, in addition to the MET inhibitor, PHA665752, as a control. (B) Immunoprecipitation of FGFR4 and immunoblotting against phosphotyrosine shows a dose-dependent inhibition of FGFR4 phosphorylation with ponatinib using the Ba/F3 TEL-FGFR4 model system. (PPTX) [file pone.0076551.s002.pptx]

## Slide 1
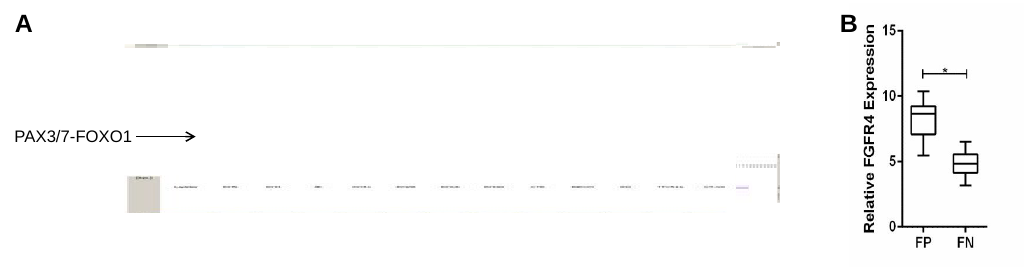

A
B
PAX3/7-FOXO1

Supplement: Figure S3 — PAX3/7-FOXO1 fusion status of cell lines and fusion-positive RMS cell lines express higher levels of FGFR4 mRNA. (A) RT-PCR with a PAX3/7-FOXO1 primer (forward: CCGACAGCAGCTCTGCCTAC and reverse: ATGAACTTGCTGTGTAGGGACAG) shows cell lines RH5, RH4, JR, RH41, RH28, and RH30 to be fusion-positive while cell lines RH18, CTR, BIRCH, RD, TTC-516, CT-10, TTC-442, and RH36 to be fusion-negative. The PAX3/7-FOXO1 band appears at 172 bp. (B) Comparison of FGFR4 mRNA expression levels between fusion-positive (FP) and fusion-negative (FN) cell lines reveals fusion-positive cell lines to express FGFR4 at higher levels than fusion-negative cell lines (p = 0.0005). FGFR4 expression was normalized to GAPDH expression. (PPTX) [file pone.0076551.s003.pptx]

## Slide 1
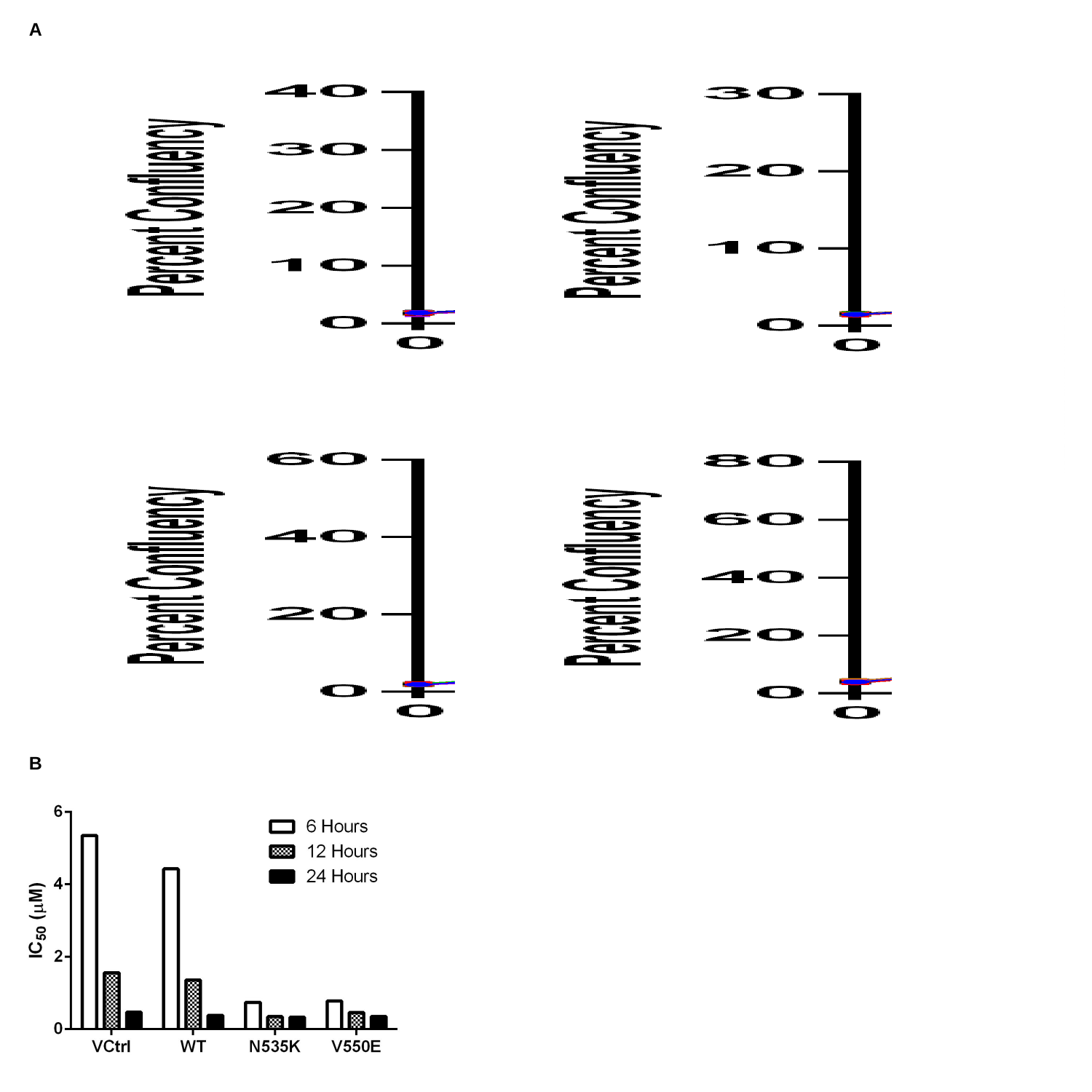

A
B

Supplement: Figure S4 — Kinetic analysis of ponatinib-induced growth inhibition for the RMS772 transductants as measured by confluency. (A) Growth curves for the RMS772 transductants illustrate differential sensitivity to ponatinib. Arrow indicates when ponatinib was added. (B) IC50 calculation at 6, 12, and 24 hours after the addition of ponatinib for RMS772 transductants shows a decrease in IC50 as time increases. (PPTX) [file pone.0076551.s004.pptx]

## Slide 1
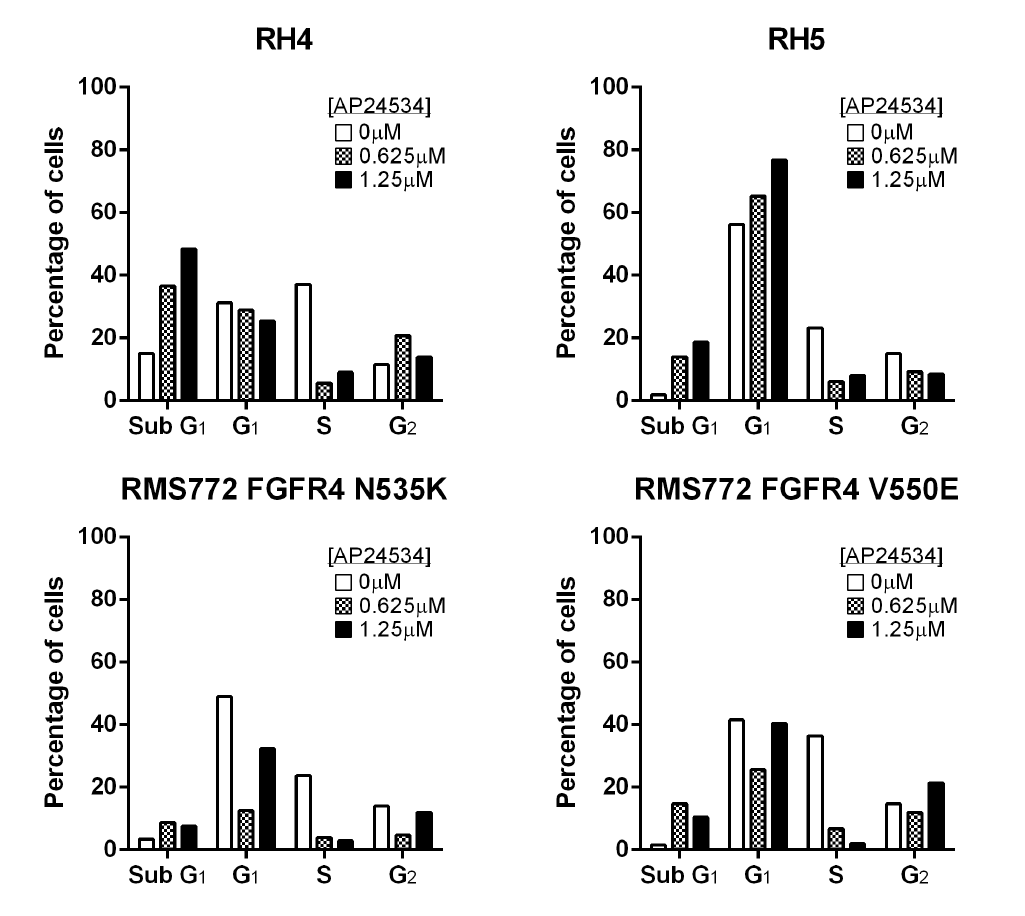

Supplement: Figure S5 — Ponatinib (AP24534) holds cell cycling at sub G1 phase and decreases time in S phase when RH4, RH5, and the two RMS772 cell lines expressing FGFR4 mutations (N535K and V550E) are treated with 0.625 and 1.25 µM ponatinib for 24 hours. (PPTX) [file pone.0076551.s005.pptx]

## Slide 1
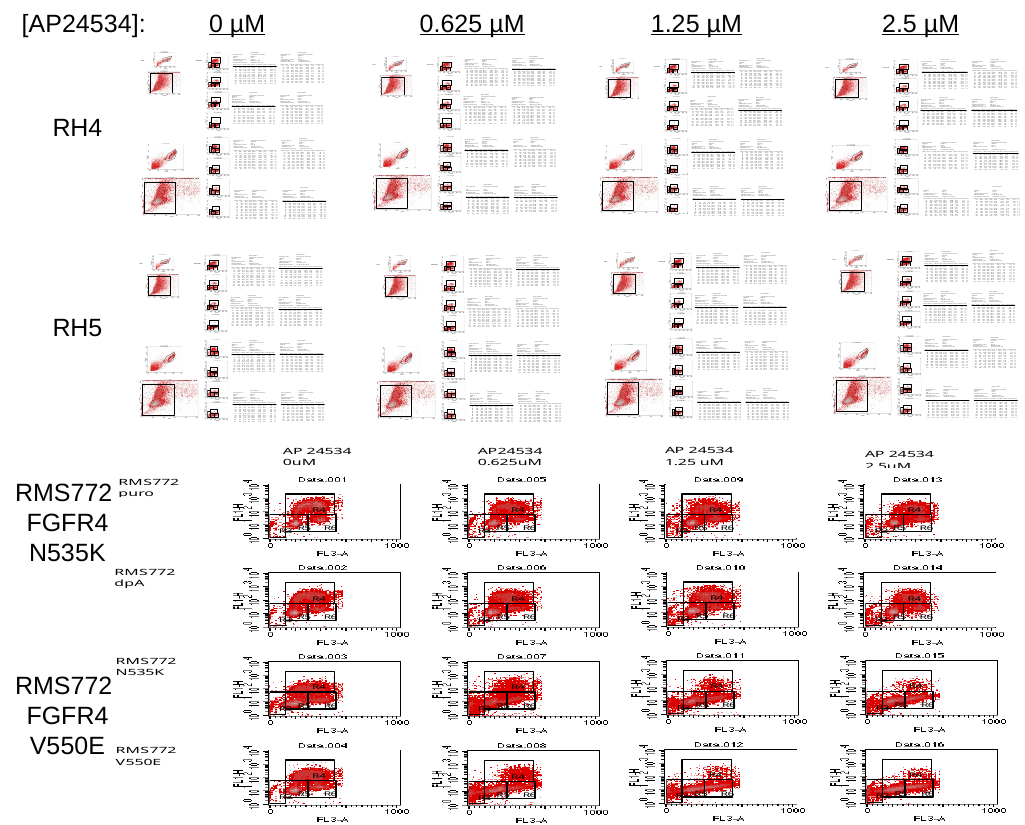

[AP24534]: 0 µM 0.625 µM 1.25 µM 2.5 µM
RH4
RH5
RMS772
FGFR4
N535K
RMS772
FGFR4
V550E

Supplement: Figure S6 — Dot plot of cell cycle flow cytometry data showing the boxes used to determine cell cycle fraction for Figure 3A and Figure S5. Each box represents a different cell cycle phase, starting with the top box and going clockwise: S phase, G2 phase, G1 phase, and subG1 phase. (PPTX) [file pone.0076551.s006.pptx]

## Slide 1
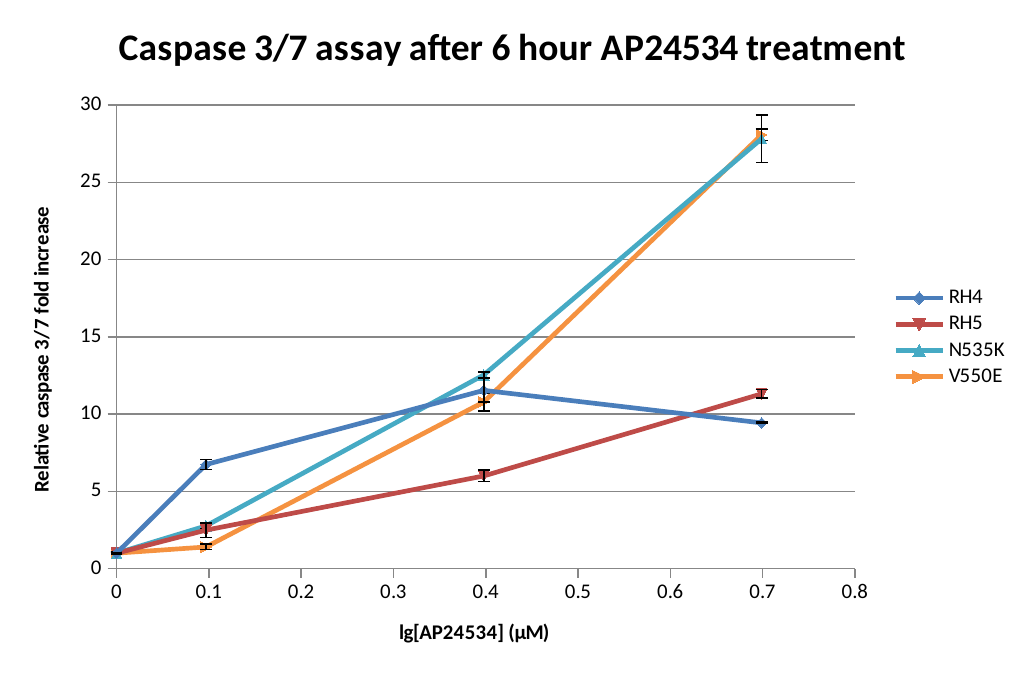

### Chart: Caspase 3/7 assay after 6 hour AP24534 treatment
| Category | | | | |
|---|---|---|---|---|

Supplement: Figure S7 — Treatment of cell lines RH4, RH5, and the two RMS772 cell lines expressing mutated FGFR4 with 0, 1.25, 2.5, and 5 µM ponatinib for 6 hours increases caspase 3/7 levels across all four cell lines. (PPTX) [file pone.0076551.s007.pptx]

## Slide 1
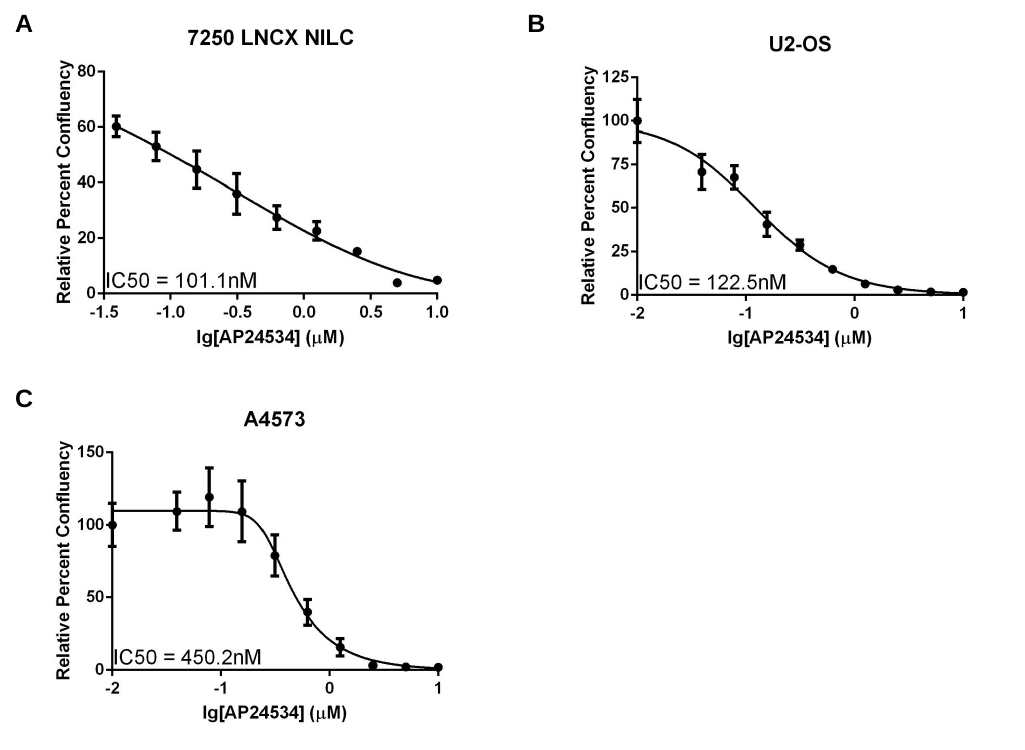

A
B
C

Supplement: Figure S8 — Fitted drug-dose response curves and calculated IC50s for cell lines (A) 7250 LNCX NILC, (B) U2-OS, and (C) A4573 after 72 hour treatment with ponatinib. (PPTX) [file pone.0076551.s008.pptx]
